# Older patients with chronic myeloid leukemia (≥65 years) profit more from higher imatinib doses than younger patients: a subanalysis of the randomized CML-Study IV

**DOI:** 10.1007/s00277-014-2041-0

**Published:** 2014-03-22

**Authors:** Ulrike Proetel, Nadine Pletsch, Michael Lauseker, Martin C. Müller, Benjamin Hanfstein, Stefan W. Krause, Lida Kalmanti, Annette Schreiber, Dominik Heim, Gabriela M. Baerlocher, Wolf-Karsten Hofmann, Elisabeth Lange, Hermann Einsele, Martin Wernli, Stephan Kremers, Rudolf Schlag, Lothar Müller, Mathias Hänel, Hartmut Link, Bernd Hertenstein, Markus Pfirrmann, Andreas Hochhaus, Joerg Hasford, Rüdiger Hehlmann, Susanne Saußele, for the German Chronic Myeloid Leukemia Study Group, and the Schweizerische Arbeitsgemeinschaft für Klinische Krebsforschung (SAKK).

**Affiliations:** 1 III. Medizinische Klinik, Universitätsmedizin Mannheim der Universität Heidelberg, Pettenkoferstrasse 22, 68169 Mannheim, Germany; 2 Institut für Medizinische Informationsverarbeitung, Biometrie und Epidemiologie, Ludwig-Maximilians-Universität, München, Germany; 3 Medizinische Klinik 5, Universitätsklinikum, Erlangen, Germany; 4 Klinik für Hämatologie, Universitätsspital, Basel, Switzerland; 5 Universitätsklinik für Hämatologie und hämatologisches Zentrallabor, Inselspital, Bern, Switzerland; 6 Klinik für Hämatologie, Onkologie und Palliativmedizin, Evangelisches Krankenhaus, Hamm, Germany; 7 Medizinische Klinik und Poliklinik II, Universitätsklinikum, Würzburg, Germany; 8 Onkologie/Hämatologie, Kantonsspital, Aarau, Switzerland; 9 Onkologisches Zentrum, Lebach, Germany; 10 Hämatologisch-Onkologische Praxis, Würzburg, Germany; 11 MVM Onkologische Schwerpunktpraxis, Leer, Germany; 12 Klinik für Innere Medizin III, Klinikum, Chemnitz, Germany; 13 Medizinische Klinik I, Westpfalz-Klinikum, Kaiserslautern, Germany; 14 Medizinische Klinik I, Klinikum Mitte, Bremen, Germany; 15 Klinik für Innere Medizin II, Universitätsklinikum, Jena, Germany

**Keywords:** Chronic myeloid leukemia, Older patients, Different imatinib dose regimens, Early applied higher imatinib dosages

## Abstract

The impact of imatinib dose on response rates and survival in older patients with chronic myeloid leukemia in chronic phase has not been studied well. We analyzed data from the German CML-Study IV, a randomized five-arm treatment optimization study in newly diagnosed BCR-ABL-positive chronic myeloid leukemia in chronic phase. Patients randomized to imatinib 400 mg/day (IM400) or imatinib 800 mg/day (IM800) and stratified according to age (≥65 years vs. <65 years) were compared regarding dose, response, adverse events, rates of progression, and survival. The full 800 mg dose was given after a 6-week run-in period with imatinib 400 mg/day. The dose could then be reduced according to tolerability. A total of 828 patients were randomized to IM400 or IM800. Seven hundred eighty-four patients were evaluable (IM400, 382; IM800, 402). One hundred ten patients (29 %) on IM400 and 83 (21 %) on IM800 were ≥65 years. The median dose per day was lower for patients ≥65 years on IM800, with the highest median dose in the first year (466 mg/day for patients ≥65 years vs. 630 mg/day for patients <65 years). Older patients on IM800 achieved major molecular remission and deep molecular remission as fast as younger patients, in contrast to standard dose imatinib with which older patients achieved remissions much later than younger patients. Grades 3 and 4 adverse events were similar in both age groups. Five-year relative survival for older patients was comparable to that of younger patients. We suggest that the optimal dose for older patients is higher than 400 mg/day. ClinicalTrials.gov identifier: NCT00055874

**Electronic supplementary material:**

The online version of this article (doi:10.1007/s00277-014-2041-0) contains supplementary material, which is available to authorized users.

## Introduction

Older patients with chronic myeloid leukemia (CML) are underrepresented in clinical trials as the median age of patients included in clinical trials is lower compared to the general population (54 years [1] vs. >60 years [2–4]). The IRIS trial [5], which led to approval of imatinib for chronic-phase (CP) CML, excluded patients over 70 years of age.

The impact of age on therapy and outcome has already been discussed in the interferon alpha (IFN) era [6]. In the imatinib era, the outcome of older patients with CML has been studied in several trials [7–13]. Most trials stratified patients by age in a group of older and a group of younger patients. Some trials included patients on different imatinib doses, but did not analyze dose effects on treatment response [8–10]. Rosti et al. reported on 284 patients in late CP CML treated with imatinib 400 mg/day. Complete cytogenetic remission (CCR) rates were lower in older patients (≥65 years) than in younger patients (<65 years) with more adverse events (AEs) in older patients, but nevertheless, overall survival (OS) was the same in both age groups [7]. Latagliata et al. analyzed 117 patients in early CP CML under imatinib treatment with 300, 400, or 800 mg/day. No significant difference in the rate of CCR was reported in older (≥65 years) compared to younger (<65 years) patients. AEs (WHO grades 3–4) were more frequent, and rates of dose reduction to ≤300 mg/day and discontinuation of imatinib were higher in older patients [8]. Cortes et al. reported on 187 patients in early CP that were treated with imatinib 400 or 600–800 mg/day. Twenty-six percent of patients were 60 years or older. With a median follow-up of 16 months, similar rates of CCR were observed in both age groups [9]. More recently, Gugliotta et al. reported similar rates of CCR and major molecular remission (MMR) in 115 patients ≥65 years among 559 patients in early CP treated with imatinib 400 or 800 mg/day [10]. In a multicenter study of high-dose imatinib in 115 newly diagnosed patients in CP, Cortes et al. reported a similar dose intensity and no difference in AEs at any severity for patients <65 and ≥65 years. MMR was achieved by 79 % of patients who received at least 90 % dose intensity (RIGHT study) [11]. Two trials addressing specifically older patients for long-term safety and tolerability of imatinib did not investigate dosage effects of imatinib [12, 13].

In contrast to the aforementioned studies, within the randomized CML-Study IV [14], we analyzed the impact of different imatinib dose regimens on response rates in imatinib-treated older CML patients in comparison to younger patients and suggest that the optimal dose for older patients is higher than 400 mg/day.

## Methods

### Study design, patients, and goals

The CML-Study IV is a five-arm randomized trial comparing imatinib 400 mg/day (IM400) vs. imatinib 800 mg/day (IM800) vs. imatinib 400 mg/day in combination with IFN vs. imatinib 400 mg/day in combination with low-dose cytarabine vs. imatinib after IFN failure in newly diagnosed BCR-ABL-positive CP CML. During a pilot phase of 3 years, only high-risk patients were assigned to imatinib 800 mg/day. In 2005, imatinib 800 mg/day was started as a full study arm [14, 15]. There was no age limit.

Primary and secondary objectives were as described previously [14, 15]. Published analyses comprised impact of remission rates on survival [14, 15], identification of prognostic factors [16, 17], and outcome of patients transplanted after imatinib pretreatment [18].

To evaluate the efficacy of imatinib in the elderly, patients randomized to IM400 and IM800 were stratified according to median age at diagnosis in western populations [3] (≥65 vs. <65 years). For all four groups, effectively administered imatinib dose, time to cytogenetic and molecular remissions, AEs by World Health Organization (WHO) grading, probabilities of progression to accelerated phase (AP) and blast crisis (BC) and OS, and causes of death were analyzed.

### Treatment

Treatment and dose adaptation were as described previously (see also legend to Table 2) [14, 15].

### Definitions and endpoints

Definitions for AP, BC, CCR, MMR, and molecular remission ≤0.01 % on the international scale (MR^4^) followed the recommendations of the European LeukemiaNet and the standardized definitions of molecular response [19, 20]. OS was defined as the time between diagnosis and death of any cause whether on or off tyrosine kinase inhibitor (TKI). All living patients were censored at the time of their last visit. In estimating the cumulative incidences (CI) of molecular or cytogenetic remissions, patients were censored at the time they received a second-generation TKI or were transplanted. Risk assignment was made by EURO [21] and EUTOS scores [22].

### Statistical analysis

Baseline characteristics were compared using the Mann–Whitney *U* test if continuous and the chi^2^ test if categorical. CI of CCR and MMR were calculated considering competing risks [23, 24] defined by AP, BC, and death. CI for AP and BC were calculated with “death without prior progression” as competing event. Comparisons were done by the Gray test [25].

OS curves were calculated by the Kaplan–Meier method and compared by the log-rank test. Relative overall survival was calculated by dividing the observed survival probabilities by the expected survival probability of the general German population matching age and sex. Analyses were according to intention-to-treat; only AEs were analyzed as treated. Level of significance was 0.05. Since this analysis was not prespecified and *p* values were not adjusted, the results have to be interpreted as exploratory. Calculations were performed with the SAS software version 9.1.3 and R 2.15.0.

### Cytogenetic and molecular analyses

Cytogenetic and molecular analyses were performed as described previously [14, 20, 26, 27].

### Ethics

The protocol followed the Declaration of Helsinki and was approved by the ethics committee of the Medizinische Fakultät Mannheim and by local ethics committees of participating centers. Written informed consent was obtained from all patients before they entered the study.

## Results

### Patients

**Fig. 1:**
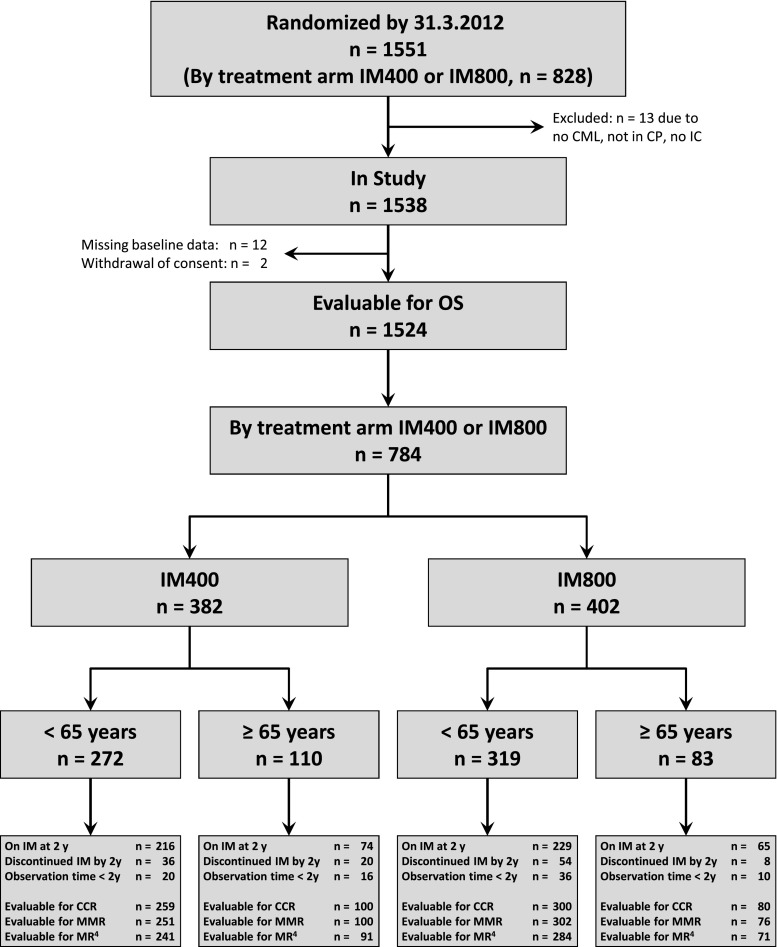
Flow diagram of randomized and evaluable patients. *n* number of patients, *IM400* imatinib 400 mg/day, *IM800* imatinib 800 mg/day, *CML* chronic myeloid leukemia, *CP* chronic phase, *IC* informed consent, *OS* overall survival, *IM* imatinib, *y* years, *CCR* complete cytogenetic remission, *MMR* major molecular remission, *MR* ^*4*^ molecular remission ≤0.01 % on the international scale

From July 2002 to March 2012, 1,551 patients were randomized, 828 of these to IM400 or IM800 (Fig. 1). A total of 784 patients were evaluable for follow-up, 382 in the IM400 and 402 in the IM800 arm. One hundred ninety-three patients were ≥65 years, 591 <65 years. Of the older patients, 110 patients were randomized to IM400 and 83 patients to IM800. The median observation time on IM800 was 50.9 months in the elderly and 50.1 months in the younger group, and on IM400, 63.0 months in the elderly and 67.6 months in the younger group. Data entry was closed on May 24, 2012.

The median age of patients was 52 years (IM400, 53 years; IM800, 51 years). Differences between the two age groups were noted in Karnofsky performance index, hemoglobin, spleen size, and white blood cell counts (Table 1).

**Table 1:** Characteristics of patients at baseline

		IM400		IM800		*p* value, IM400/IM800 combined
Age (years), (*n*)		<65 (272)	≥65 (110)	<65 (319)	≥65 (83)	<65 vs. ≥65
Age (years), median (range)		48 (16–64)	70 (65–88)	46 (18–64)	69 (65–85)	–
Sex female (%)		38	45	41	41	ns
Karnofsky index (%)	0–85	11	16	10	18	0.03
	90–95	31	38	31	30	0.03
	100	58	46	59	52	0.03
Hemoglobin (g/dl), median (range)		12.1 (4.9–17.5)	12.8 (6.4–16.2)	12.1 (4.7–19.1)	12.4 (6.5–15.7)	<0.01
White blood cell count ×10^9^/l, median (range)		81.6 (5.7–574)	58.0 (6.6–582)	93.8 (2.6–554)	43.8 (5.1–570)	<0.01
Platelets ×10^9^/l, median (range)		373 (89–2,419)	390 (58–2,337)	388 (39–2,582)	400 (88–2,716)	ns
Spleen size (cm below costal margin), median (range)		2 (0–28)	0 (0–16)	2 (0–30)	0 (0–23)	<0.01
EURO score (%)	Low	45	11	45	12	nd^a^
	Intermediate	42	77	42	72	nd^a^
	High	13	12	13	16	nd^a^
EUTOS score (%)	Low	87	91	85	86	ns
	High	13	9	15	14	ns

*n* number of patients, *IM400* imatinib 400 mg/day, *IM800* imatinib 800 mg/day, *ns* not significant, *nd* not done

^a^Since the EURO score depends on age by definition, no test was conducted

The median dose per day for the entire observation time was lower for older patients on IM800 (421 mg/day for patients ≥65 years vs. 556 mg/day for patients <65 years) with the highest median dose in the first year (466 mg/day for patients ≥65 years vs. 630 mg/day for patients <65 years), but not different for both age groups on IM400 (400 mg/day each). The dynamics of dose adaptation were analyzed in 3-month periods during the first 24 months of treatment for all four patients groups (Table 2). Cutoff values of 390 and 790 mg/day have been chosen to distinguish between the patients that really changed dose and those that only discontinued imatinib for one or two days, e.g., due to AEs. The 47.5 % of younger patients and 18.4 % of older patients on IM800 received an imatinib dosage between 790 and 800 mg/day in the second 3-month period with the highest dose in older patients at 494 mg/day in the second 3-month period. Thereafter, the dose decreased to 400 mg/day by months 6–9. In younger patients, the highest median dosage on IM800 was 773 mg/day in the second 3-month period, which decreased to 600 mg/day by months 9–12.

**Table 2:** Dose adaptation of imatinib during the first 24 months

		IM400		IM800	
Age (years)		<65	≥65	<65	≥65
Months	Doses				
	Median (mg/day)				
0–3		400	400	563	472
3–6		400	400	773	494
6–9		400	400	629	400
9–12		400	400	600	400
12–15		400	400	600	400
15–18		400	400	600	400
18–21		400	400	600	400
21–24		400	400	600	400
	<390 mg/day (%)				
0–3		3.5	4.9	1.3	0.0
3–6		6.4	4.0	4.3	2.6
6–9		5.8	3.2	3.6	0.0
9–12		4.9	3.4	2.2	0.0
12–15		3.4	4.7	2.0	1.5
15–18		3.2	3.7	1.3	1.6
18–21		2.8	3.8	2.2	0.0
21–24		2.5	4.0	2.4	0.0
	390–410 mg/day (%)				
0–3		94.6	94.1	21.8	39.0
3–6		91.0	95.0	20.8	36.8
6–9		89.2	95.7	32.2	58.0
9–12		87.7	94.3	41.1	62.1
12–15		89.0	92.9	41.7	69.1
15–18		87.8	87.7	43.0	68.9
18–21		88.2	88.8	41.6	70.7
21–24		86.4	86.7	44.5	71.7
	>410–599 mg/day (%)				
0–3		1.5	0.9	41.6	36.6
3–6		1.1	0.0	11.6	23.7
6–9		3.1	1.1	9.2	8.7
9–12		4.5	2.3	4.5	12.1
12–15		3.8	0.0	4.0	3.4
15–18		2.7	6.2	3.4	3.3
18–21		2.4	3.8	3.1	3.5
21–24		4.0	2.7	1.0	3.8
	600–790 mg/day (%)				
0–3		0.4	0.0	14.5	8.5
3–6		0.8	0.0	12.9	18.4
6–9		1.5	0.0	7.1	15.9
9–12		2.1	0.0	3.4	10.6
12–15		0.0	2.4	4.4	8.8
15–18		4.1	0.0	9.8	6.6
18–21		4.2	0.0	9.7	8.6
21–24		4.0	0.0	8.1	9.4
	>790–800 mg/day (%)				
0–3		0.0	0.0	18.8	15.9
3–6		0.8	1.0	47.5	18.4
6–9		0.4	0.0	44.3	17.4
9–12		0.8	0.0	44.8	15.2
12–15		0.0	0.0	41.7	16.2
15–18		2.3	1.2	42.6	19.7
18–21		2.4	3.8	42.9	17.2
21–24		3.0	4.0	43.5	0.0

Initial treatment for IM400 was imatinib 400 mg/day once daily. In case of suboptimal response, a dose increase to 600 or 800 mg/day was permitted. For IM800, the full 800 mg dose was given after a 6-week run-in period with imatinib 400 mg/day to avoid excessive cytopenias. The dose could then be reduced according to tolerability for maximum patients’ adherence and to avoid clinical risks

*IM400* imatinib 400 mg/day, *IM800* imatinib 800 mg/day, *%* percent of patients who were still on imatinib at the respective time points and received these doses

### Comparison of responses

**Fig. 2:**
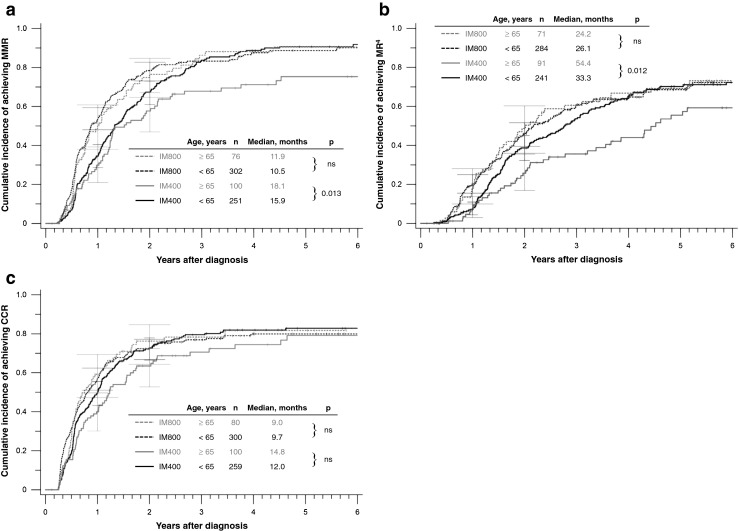
Molecular and cytogenetic remissions according to treatment groups. **a** Cumulative incidences of MMR, **b** cumulative incidences of MR^4^, and **c** cumulative incidences of CCR. *MMR* major molecular remission, *MR* ^*4*^ molecular remission ≤0.01 % on the international scale, *CCR* complete cytogenetic remission, *n* number of patients, *IM400* imatinib 400 mg/day, *IM800* imatinib 800 mg/day, *ns* not significant

If treated with IM800, older patients achieved MMR and MR^4^ as fast as younger patients (Fig. 2a, b). Median times to MMR were 11.9 vs. 10.5 months, respectively. Median times to MR^4^ were 24.2 vs. 26.1 months, respectively. In contrast, if treated with IM400, older patients achieved MMR and MR^4^ significantly later than younger patients (MMR *p* = 0.013; Fig. 2a; MR^4^ *p* = 0.012; Fig. 2b). Median times to MMR were 18.1 vs. 15.9 months, and to MR^4^ 54.4 vs. 33.3 months, respectively. Regarding CCR, median times to CCR on IM800 were 9.0 vs. 9.7 months, and on IM400 14.8 vs. 12.0 months, respectively. The difference between age groups on IM400 did not reach significance (Fig. 2c).

### AEs

One hundred sixty-nine patients ≥65 years and 542 patients <65 years were evaluated for AEs during the initial 24 months. There was no significant difference between age groups for higher-grade (WHO grades 3 and 4) hematologic AEs. Leukocytopenia (all grades) in patients treated on IM800 and neurological AEs (all grades) in patients treated on IM400 were significantly less frequent in older patients (*p* = 0.009 and *p* = 0.03, respectively). Some higher-grade non-hematologic AEs were significantly more frequent in older than in younger patients (IM400: dermatologic AEs, *p* = 0.01; IM800: infections, *p* = 0.03) (Table 3).

**Table 3:** Adverse events during the initial 24 months

	IM400		IM800	
Age (years), (*n*)	<65 (254^a^)	≥65 (96^a^)	<65 (288^a^)	≥65 (73^a^)
Adverse events				
WHO grades 1–4 (%)				
Anemia^b^	51.4	61.4	53.1	54.9
Leukocytopenia^c^	56.7	58.3	64.7	48.0
Thrombocytopenia^d^	36.9	34.5	35.6	32.9
Myalgia/arthralgia^e^	19.0	11.7	28.9	20.8
Dermatologic AEs^e^	12.6	14.9	27.5	33.3
Edema^e^	24.9	24.5	40.1	45.8
Gastrointestinal AEs^e^	26.1	23.4	48.2	47.2
Neurological AEs^e^	15.4	6.4	16.9	20.8
Infection^e^	6.3	4.3	12.0	15.3
Fatigue^e^	14.2	8.5	19.7	15.3
Allergy/immunology^e^	1.2	4.3	2.5	0
Constitutional symptoms^e^	5.9	3.2	14.8	12.5
Other^e^	31.2	30.9	42.7	45.2
WHO grades 3 and 4 (%)				
Anemia^f^	3.8	6.4	5.1	7.0
Leukocytopenia	1.6	3.1	5.2	5.5
Thrombocytopenia	3.9	4.2	7.7	8.2
Myalgia/arthralgia^e^	2.8	1.1	2.5	1.4
Dermatologic AEs^e^	0.4	5.4	2.8	2.8
Edema^e^	1.2	0	1.4	5.6
Gastrointestinal AEs^e^	2.0	1.1	3.2	4.8
Neurological AEs^e^	2.0	0	2.5	4.2
Infection^e^	0.8	0	2.5	8.3
Fatigue^e^	0.4	0	2.5	1.4
Allergy/immunology^e^	0	1.1	0.4	0
Constitutional symptoms^e^	0.4	0	0.7	0
Other^e^	6.3	9.6	11.6	12.5

*n* number of patients, *IM400* imatinib 400 mg/day, *IM800* imatinib 800 mg/day, *WHO* World Health Organization, *%* percent of patients who had the described adverse events, *AEs* adverse events

^a^Number of patients for whom an analysis was available. A total of 73 out of 784 patients were not evaluable due to short observation time (<24 months) and being alive

^b^In addition, 276 patients were not evaluable due to anemia grades 1–4 at baseline

^c^In addition, 5 patients were not evaluable due to leukocytopenia grades 1–4 at baseline

^d^In addition, 35 patients were not evaluable due to thrombocytopenia grades 1–4 at baseline

^e^In addition, 8 patients were not evaluable due to missing information about non-hematologic adverse events

^f^In addition, 32 patients were not evaluable due to anemia grades 3–4 at baseline

### Progression and survival

**Fig. 3:**
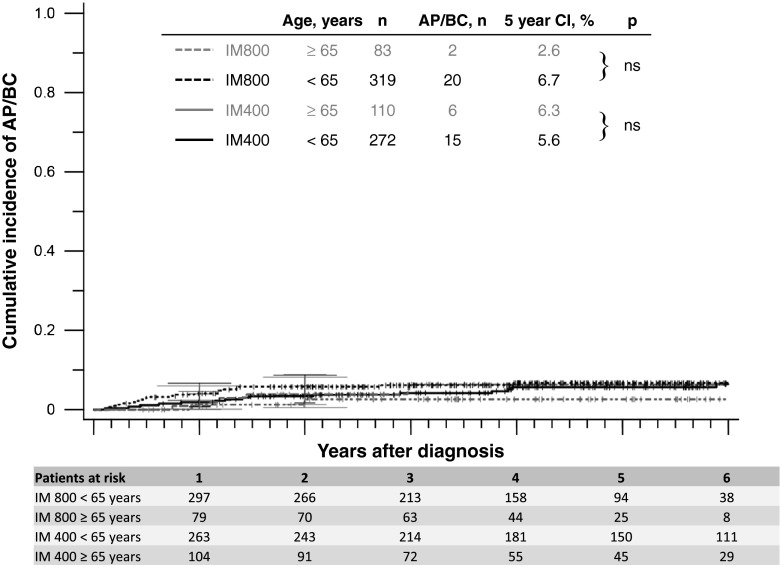
Progression to AP and BC according to treatment groups. *AP* accelerated phase, *BC* blast crisis, *n* number of patients, *CI* cumulative incidence, *IM400* imatinib 400 mg/day, *IM800* imatinib 800 mg/day, *ns* not significant

There was no difference between age groups in probabilities of progression to AP or BC in an analysis according to treatment groups (Fig. 3). Five-year OS for patients ≥65 years was 78.2 % (IM400) and 87.5 % (IM800), and for patients <65 years 92.8 % (IM400) and 92.5 % (IM800). Taking into account the German population adjusted for age and sex [28], 5-year relative survival was 90.7 % for IM400 and 100.8 % for IM800, respectively, for the older patients and 94.9 % and 94.4 %, respectively, for the younger patients. In the elderly, death due to second malignancies was more frequent than death due to progression (Table 4).

**Table 4:** Causes of death

	IM400		IM800	
Age (years), (*n*)	<65 (272)	≥65 (110)	<65 (319)	≥65 (83)
Total deaths (*n*)	23	21	19	6
Causes (*n*)				
Progression to AP/BC	8	4	9	1
Transplantation related	3	0	4	0
Infection in CP	2	0	2	1
Secondary malignancy	1	8	1	2
Bleeding	1	0	1	0
Cardiopulmonary	2	2	1	1
Renal insufficiency	0	2	0	1
Thromboembolic/ischemic (not cardiac)	0	1	1	0
Suicide	0	1	0	0
Others	3	0	0	0
Unknown	3	3	0	0

*n* number of patients, *IM400* imatinib 400 mg/day, *IM800* imatinib 800 mg/day, *AP* accelerated phase, *BC* blast crisis, *CP* chronic phase

## Discussion

This is the first report that analyzes the effect of different imatinib dose regimens in older vs. younger patients with CML. The most important finding of our analysis is that older patients on IM800 had no delay in reaching MMR and MR^4^, as this was the fact with standard-dose imatinib where MMR and MR^4^ were achieved significantly later than in younger patients. We conclude that the superiority of the response rates to IM800 was more pronounced in the older than in the younger group. This effect is remarkable as the median dose for older patients on IM800 was lower than that of younger patients and only moderately higher than in older patients on IM400. The result is in line with previous reports of this study that superior cytogenetic and molecular remission rates were reached in patients with IM800 [14]. We think that this finding is important, since superior molecular remission rates have been shown to correlate with better survival [14, 15].

To avoid higher-grade AEs on IM800, imatinib was adapted to tolerability in both age groups. Dose reductions were higher in older patients, although AEs grades 1–4 occurred not more frequently than in younger patients. We hypothesize that dose reductions in older patients were done low-threshold compared to younger patients, to avoid clinical risks in a frailer population. The observed differences in AEs might be random. A similar dose intensity and no difference in AEs was reported in high-dose imatinib therapy for patients <65 and ≥65 years by Cortes et al. [11], whereas grades 3–4 hematologic and non-hematologic AEs were reported to be more frequent in older patients with early CP CML by Latagliata et al. [8] and in late CP treated with imatinib 400 mg/day by Rosti et al. [7].

It should be mentioned that most non-hematologic AEs occurred more often in the IM800 arm, independent of age, but since grades 3 and 4 AEs were similar between IM400 and IM800, this appears tolerable with regard to a potentially better outcome.

Given the observation that the effect of higher imatinib dosages applied early in the course of treatment is more pronounced in older than in younger patients, this could explain the difference between results of this study and another randomized trial of imatinib 400 vs. 800 mg/day [29], since in the other study, the median age of the study population was lower (47 vs. 52 years in our study) and patients older than 75 years were excluded, resulting in a lower number of patients ≥65 years (15.5 % (personal written communication, C. Piccolo, Novartis, November 18, 2013) vs. 24.6 % in our study).

Since the median dosage for patients on the IM400 arm was 400 mg/day for both age groups and the proportion of patients who received doses <390 mg/day was similar, the significantly later achievement of MMR and MR^4^ in older patients on IM400 cannot be explained by non-adherence to the prescribed medication and a lower than 400 mg dosage. The baseline characteristics beyond age seem to have no influence, but the proportion of patients with lower Karnofsky index was significantly higher in older patients.

To compare survival between age groups, the German population adjusted for age and sex [28] was taken into account. OS was reduced in older compared to younger patients due to a generally reduced life expectancy of older people, whereas the 5-year relative survival of older patients was comparable with that of younger patients. Nevertheless, it is important to note that a bias in favor of the study patients is likely. The relative survival estimates may be too optimistic, since the exclusion criteria of CML-Study IV prevented the participation of some of the frailest patients, e.g., those with other neoplasias in need of treatment or with conditions preventing study compliance and thus with a supposedly reduced life expectancy. This would explain the better survival in older patients on IM800 (100.8 % at 5 years) than in the general population.

In the pre-imatinib era, older age has been a poor prognostic factor in CML [21, 30]. In the EUTOS score, which was based on patients treated with imatinib, age is not included any more. Since the EUTOS score refers to the endpoint CCR at 18 months [22], it has to be seen in the future whether age is still an important risk factor for survival in patients with CML under imatinib.

In conclusion, in older patients, higher molecular response rates similar to younger patients are achievable with higher imatinib doses applied early in the course of treatment, in contrast to standard-dose imatinib. It seems that the optimal daily dose for patients with CP CML is higher than 400 mg, irrespective of age.

## Electronic supplementary material

Below is the link to the electronic supplementary material.

ESM 1 A list of the German Chronic Myeloid Leukemia Study Group and the SAKK (participants of the CML-Study IV) appears in a supplemental Appendix in the online article. (PDF 85.4 kb)
