# Supplementary material for: Older patients with chronic myeloid leukemia (≥65 years) profit more from higher imatinib doses than younger patients: a subanalysis of the randomized CML-Study IV
Source: Ann Hematol. 2014 Mar 22;93(7):1167–76. doi: 10.1007/s00277-014-2041-0 (PMC4050299; doi:10.1007/s00277-014-2041-0)
Supplement: Supplementary file 1 — A list of the German Chronic Myeloid Leukemia Study Group and the SAKK (participants of the CML-Study IV) appears in a supplemental Appendix in the online article. (PDF 85.4 kb) [file 277_2014_2041_MOESM1_ESM.pdf]

German CML-Study Group and SAKK
(Participants of the CML-Study IV)

Ort	Institution	Vorname	Nachname
Aarau (Switzerland)	Kantonsspital	Martin	Wernli
		Mario	Bargetzi
Amberg	Klinikum St. Marien	Ludwig	Fischer von Weikersthal
		Volker	Groß
Ansbach	Ambulantes Onkologie Zentrum	Sebastian	Müller
		Markus	Hahn
Augsburg	Klinikum	Günter	Schlimok
Aurich	Gemeinschaftspraxis für Hämatologie und Onkologie	Dietmar	Reichert
		Jan	Janssen
Bad Friedrichshall	Klinikum am Plattenwald	Jürgen	Furkert
		Thomas	Mandel
Bad Hersfeld	Klinikum	Peter-J.	Majunke
		Roland	Paliege
Bad Saarow	Helios Klinikum	Peter	Reichert
		Harald	Fuss
Baden-Baden	ACURA Kliniken	Kai	Neben
		Hans-Jürgen	Staiger
Basel (Switzerland)	Universitätsspital	Dominik	Heim
		Alois	Gratwohl
		André	Tichelli
Bergisch Gladbach	Vinzenz Pallotti Hospital Bensberg	Stefan	Korsten
		Dirk	Henesser
Berlin	Charité - Universitätsmedizin	Philipp	Le Coutre
		Renate	Arnold
	Helios Klinikum Buch	Wolf-D.	Ludwig
		Richard	Ratei
		Christian	Teutsch
	MVZ Hämatologie Onkologie Tempelhof	Friedrich	Ludwig
		Antje	Urmersbach

German CML-Study Group and SAKK
(Participants of the CML-Study IV)

Berlin	Onkologische Praxis am Gesundbrunnen	Ilona	Blau
		Helgrid	Ihle
	Onkologische Schwerpunktpraxis Berlin-Mitte	Christian	Sperling
		Claudia	Schelenz
	Onkologische Schwerpunktpraxis Tiergarten	Antje	Koschuth
		Dorothea	Kingreen
	Praxis für Innere Medizin	Jörg	Heßling
	St. Hedwig-Krankenhaus	Karl-M.	Derwahl
		Bernd	Oldenkott
		Hans-J.	Englisch
		Friedrich	Ludwig
	Universitätsklinikum Benjamin Franklin der FU	Eckhard	Thiel
		Thomas	Burmeister
		Michael	Notter
	Vivantes Klinikum Neukölln	Maike	de Wit
		Winfried	Rothaug
		Gerd	Büschel
	Vivantes Krankenhaus Am Urban	Jörg	Beyer
		Elmar	Dahmen
Bern (Switzerland)	Swiss Group for Clincial Cancer Research (SAKK)	Christine	Biaggi
	Inselspital, Universitätsspital	Bernhard	Lämmle
		Dorothea	Friess
		Gabriela	Baerlocher
		Elisabeth	Oppliger Leibundgut
		Andreas	Tobler
Bielefeld	Onkologische Schwerpunktpraxis	Marianne	Just
		Erhardt	Schäfer
Bochum	Augusta-Kranken-Anstalt	Dirk	Behringer
		Michael	Brandt
	Universitätsklinik Knappschaftskrankenhaus	Wolff	Schmiegel
		Christian	Teschendorf

German CML-Study Group and SAKK
(Participants of the CML-Study IV)

Bonn	Gemeinschaftspraxis für Hämatologie und Onkologie	Hans A.	Vaupel
		Walter	Verbeek
	Johanniter-Krankenhaus	Yon-Dschun	Ko
		Stephan	Weidenhöfer
	Universitätsklinikum	Tilman	Sauerbruch
		Corinna	Hahn-Ast
		Viktor	Janzen
		Ingo	Schmidt-Wolf
Bottrop	Knappschaftskrankenhaus	Guido	Trenn
		Margret	van der Linde
Brandenburg	Städtisches Klinikum	Wilfried	Pommerien
		Lüder	Fritz
Braunschweig	Klinikum	Jürgen	Krauter
		Florian	Lordick
		Gisela	Fritsch
Bremen	DIAKO	Karl-H.	Pflüger
		Christoph	Diekmann
		Johannes	Kullmer
	Gemeinschaftspraxis für Hämatologie und Onkologie	Gabriele	Doering
		Heike	Munzinger
	Klinikum Mitte	Bernd	Hertenstein
		Astrid	Peyn
Brno (Czech Republic)	The University Hospital	Jiri	Mayer
		Daniela	Zácková
		Jitka	Kujickova
Brühl	Onkologische Schwerpunktpraxis	Sebastian	Stier
Bünde	Lukas Krankenhaus	Bernd	Wejda
		Ferdinand	Möller-Faßbender
Chemnitz	Klinikum	Mathias	Hänel
		Anke	Morgner
		Regina	Herbst

German CML-Study Group and SAKK
(Participants of the CML-Study IV)

Coburg	Klinikum	Werner	Matek
		Christof	Lamberti
	Onkologische Schwerpunktpraxis	Thomas	Zöller
Datteln	St. Vincenz Krankenhaus	Bernhard	Koch
Daun	Krankenhaus Maria Hilf	Thomas	Marth
		Adalbert	Henzel
Deggendorf	DONAUISAR Klinikum	Siegfried	Wagner
		Elisabeth	Woska
Delmenhorst	Städtische Kliniken	Frank	Neumann
Dernbach	Praxis für Hämatologie und Onkologie	Matthias M.	Hoffknecht
Dresden	Gemeinschaftspraxis Hämatologie - Onkologie	Thomas	Illmer
		Thomas	Wolf
	Universitätsklinikum Carl Gustav Carus	Gerhard	Ehninger
		Alexander	Kiani
		Uwe	Platzbecker
Duisburg	HELIOS St. Johannes Klinik	Carlo	Aul
		Curd-D.	Badrakhan
		Aristoteles	Giagounidis
Düren	Krankenhaus	Michael	Flaßhove
		Felicitas	Henneke
		Thomas	Moritz
Ehingen	Internistische Praxisgemeinschaft	Martin	Simon
Emden	Onkologische Schwerpunktpraxis Leer	Lothar	Müller
Erkelenz	Praxis für Innere Medizin, Hämatologie und Onkologie	Rudolf	Janz
Erlangen	Internistische Schwerpunktpraxen	Michael	Eckart
		Babette	Häcker
	Universitätsklinikum	Dorit	Rech
		Andreas	Mackensen
		Stefan W.	Krause
Eschweiler	St. Antonius Hospital	Peter	Staib
		Frank	Schlegel
		Kristin	Wätzig

German CML-Study Group and SAKK
(Participants of the CML-Study IV)

Essen	Hämatologisch-Onkologische Gemeinschaftspraxis	Roland	Rudolph
	Kliniken Süd	Mohammed	Wattad
		Franz-Karl	Baur
		Wolfgang	Heit
	Universitätsklinikum	Dietrich W.	Beelen
		Andreas	Hüttmann
		Jürgen	Novotny
		Rudolf	Trenschel
Ettlingen	Praxis für Innere Medizin	Albrecht	Lindemann
Euskirchen	Praxiskooperation	Dominik	Linck
Frankfurt am Main	Krankenhaus Nordwest	Elke	Jäger
		Salah-E.	Al-Batran
	Universitätsklinikum	Oliver G.	Ottmann
		Hubert	Serve
Freiburg	Onkologische Praxis	Tobias	Reiber
	Praxis für interdisziplinäre Onkologie und Hämatologie	Dieter	Semsek
	Universitätsklinikum	Cornelius	Waller
		Alexander	Kühnemund
Fulda	Klinikum	Heinz-G.	Hoeffkes
Garmisch-Partenkirchen	Klinikum	Helmut	Lambertz
		Lothar	Schulz
Geilenkirchen	Praxis für Innere Medizin	Kian	Tajrobehkar
Germering	Gemeinschaftspraxis für Hämatologie und Onkologie	Johann	Mittermüller
Gießen	Universitätsklinikum	Mathias J.	Rummel
		Alexander	Burchardt
		Hans	Pralle
Goch	Wilhelm-Anton-Hospital	Volker	Runde
		Mathias	Kleiß
		Jörn	Westheider
Goslar	Onkologische Kooperation Harz	Andreas	Hoyer
		Hans W.	Tessen
		Alexandra	Hesse

German CML-Study Group and SAKK
(Participants of the CML-Study IV)

Göttingen	Universitätsmedizin	Lorenz	Trümper
		Claudia	Binder
Greifswald	Universitätsklinikum	Christian-A.	Schmidt
		Frank	Schüler
		Gottfried	Dölken
		Carsten	Hirt
Gummersbach	Kreiskrankenhaus	Markus	Sieber
Güstrow	Onkologische Schwerpunktpraxis	Henning	Eschenburg
		Stefan	Wilhelm
Gütersloh	Onkologische Schwerpunktpraxis	Reinhard	Depenbusch
		Siegfried	Rösel
Hagen	St.-Marien-Hospital	Hans-W.	Lindemann
		Hartmut	Eimermacher
Halle/Saale	Hämatologisch- onkologische Gemeinschaftspraxis	Claudia	Spohn
		Regina	Moeller
	Universitätsklinikum	Hans-H.	Wolf
		Hans-J.	Schmoll
Hamburg	Allgemeines Krankenhaus Altona	Dietrich	Braumann
		Petra	Hoelzer
Hamburg	Asklepios Klinik St. Georg	Norbert	Schmitz
		Maike	Nickelsen
	Hämatologisch-onkologische Praxis Altona	Ulrich R.	Kleeberg
		Erik	Engel
		Tiina	Haatanen
		Wiebke	Hollburg
		Dieter	Platz
	Hämatologisch-Onkologisches Zentrum Ost	Hubertus	Köster
	Universitätsklinikum Eppendorf	Carsten	Bokemeyer
		Philippe	Schafhausen
		Dieter	Hossfeld

German CML-Study Group and SAKK
(Participants of the CML-Study IV)

Hamm	Evangelisches Krankenhaus	Elisabeth	Lange
		Jörg	Schubert
	Hämatologische Gemeinschaftspraxis	Heinz	Weischer
		Albert	Grote-Metke
		Britta	Bechtel
	St. Marien-Hospital	Heinz A.	Dürk
		Marc	Hemeier
Hannover	Klinikum Siloah	Hartmut H.	Kirchner
		Markus	Sosada
	Medizinische Hochschule Hannover	Arnold	Ganser
		Brigitte	Schlegelberger
		Dietrich	Peest
Heidelberg	Universitätklinikum	Anthony D.	Ho
		Eike C.	Buß
		Sarah	Rohlfing
		Jolanta	Dengler
Heidenheim	Praxis für Innere Medizin	Volker	Petersen
Heilbronn	Schwerpunktpraxis Onkologie	Piotr	Porowski
Herford	Klinikum	Stephan	Bildat
		Johann G.	Lange
Herne	Praxisklinik	Lars	Hahn
Herrsching-Ammersee	Praxis für Hämatologie und Onkologie	Hermann	Dietzfelbinger
Hersbruck	Onkologische Schwerpunktpraxis	Wolfram	Gröschel
Hildesheim	Onkologie im Medicinum	Bernd	Sievers
		Werner	Freier
	St. Bernward Krankenhaus	Ulrich	Kaiser
		Andrea	Bartholomäus
Homburg/Saar	Universitätsklinik	Michael	Pfreundschuh
Idar-Oberstein	Klinikum	Eva	Römer
		Thomas	Herrmann
		Axel	Fauser
		Maritza L.	Valverde

German CML-Study Group and SAKK
(Participants of the CML-Study IV)

Ingolstadt	Klinikum	Josef	Menzel
Iserlohn	MVZ am Ev. Krankenhaus Bethanien	Michael	Kemmerling
		Jürgen	Kemper
Jena	Universitätsklinikum	Andreas	Hochhaus
		Paul	La Rosée
Kaiserslautern	Hämatologisch-Onkologische Praxis	Richard	Hansen
		Manfred	Reeb
	Westpfalz-Klinikum	Hartmut	Link
		Stefan	Mahlmann
Karlsruhe	St. Vincentius-Kliniken	Jörg	Mezger
		Michael	Schatz
	Städtisches Klinikum	Martin	Bentz
		Sibylla	Wilhelm
		Margarethe	Schmier
Kempten	Klinikum Oberallgäu	Otto	Prümmer
		Juergen	Gatter
Kiel	Universitätsklinikum Schleswig-Holstein	Michael	Kneba
		Svenja	Neumann
		Uwe	Strack
		Robert	Schoch
Koblenz	Praxis für Hämatologie und Onkologie	Jochen	Heymanns
Köln	Praxis für Hämatologie und Internistische Onkologie	Hans T.	Steinmetz
		Stephan	Schmitz
		Kai	Severin
	Uniklinik	Christof	Scheid
Krefeld	Klinikum	Manfred	Planker
		Thomas	Frieling
	Onkologische Gemeinschaftspraxis	Andre	Lollert
		Michael	Neise
Kronach	Onkologische Schwerpunktpraxis	Martina	Stauch
Landau	Vinzentiuskrankenhaus	Martin	Schröder
		Ulrich	Karbach

German CML-Study Group and SAKK
(Participants of the CML-Study IV)

Landshut	Internistische Gemeinschaftspraxis	Ursula	Vehling-Kaiser
		Doris	Greif
	Klinikum	Barbara	Kempf
		Wolfgang	März
Lebach	Onkologisches Zentrum	Stephan	Kremers
Leer	Kreiskrankenhaus	Georg	Köchling
	Onkologische Schwerpunktpraxis	Lothar	Müller
Lemgo	Klinikum	Frank	Hartmann
		Ulrich	Weiß
		Helmut	Middeke
Limburg	St. Vincenz Krankenhaus	Thomas	Neuhaus
		Heike	Martin
Lübeck	Sana Kliniken	Sebastian	Fetscher
		Jan	Schmielau
Lüdenscheid	Klinikum	Gerhard	Heil
	Praxis für Hämatologie/Onkologie	Dietrich	Kämpfe
Ludwigshafen	Klinikum Ludwigshafen	Michael	Uppenkamp
		Burkhard	Weiß
Lüneburg	Onkologische Schwerpunktpraxis	Bernhard	Goldmann
		Peter	Heinkele
Luzern (Switzerland)	Kantonsspital	Peter	Thum
		Walter	Wuillemin
		Michael	Gregor
Mainz	Universitätsmedizin	Matthias	Theobald
		Thomas	Fischer
		Simone	Thomas
Mannheim	Onkologie Praxis	Manfred	Hensel
		Christoph	Plöger
		Dieter	Schuster
		Jürgen	Brust
	Praxis für Innere Medizin	Udo	Hieber

German CML-Study Group and SAKK
(Participants of the CML-Study IV)

Mannheim	Universitätsmedizin	Wolf-K.	Hofmann
		Benjamin	Hanfstein
		Martin	Müller
		Susanne	Saußele
		Rüdiger	Hehlmann
Marburg	Universitätsklinikum	Andreas	Neubauer
		Andreas	Burchert
Minden	Johannes Wesling Klinikum	Martin	Griesshammer
		Hans-J.	Tischler
	Onkologische Praxis	Martin	Becker
Mönchengladbach	Krankenhaus St. Franziskus	Ullrich	Graeven
		Christiane	Lange
Muhr am See	Onkologische Schwerpunktpraxis	Bernhard	Göttler
		Gerhard	Schmidt
Mülheim	Praxis für Innere Medizin	Christian	Lunscken
München	Onkologische Schwerpunktpraxis	Siegfried	Völkl
	Hämatologische Praxisgemeinschaft	Hans-D.	Schick
		Burkhard	Schmidt
	Hämatologische Schwerpunktpraxis	Helmut	Hitz
	Klinikum der Ludwig-Maximilians-Universität	Karsten	Spiekermann
		Hans-J.	Kolb
		Wolfgang	Hiddemann
	Klinikum Dritter Orden	Stephan	Weidenhöfer
		Peter	Weidinger
	MLL Münchner Leukämielabor	Torsten	Haferlach
		Claudia	Haferlach
		Susanne	Schnittger
	Onkologische Schwerpunktpraxis	Oliver	Stötzer
	Praxis für Innere Medizin und HämatoOnkologie	Clemens	Scheidegger
	Städtisches Klinikum Harlaching	Ludwig	Lutz
		Marcus	Hentrich

German CML-Study Group and SAKK
(Participants of the CML-Study IV)

München	Städtisches Klinikum Schwabing	Christoph	Nerl
		Clemens	Wendtner
		Christof	Fischer
Münster	Gemeinschaftspraxis für Hämatologie und Onkologie	Jürgen	Wehmeyer
	Universitätsklinikum	Wolfgang E.	Berdel
		Eva	Schmidt
		Steffen	Koschmieder
		Adriane	Koppele
Mutlangen	Stauferklinikum Schwäbisch Gmünd	Holger	Hebart
		Armin	Snaga
Neumarkt	Onkologische Schwerpunktpraxis	Ekkehart	Ladda
		Marcus	Gnad
Neunkirchen	Onkologische Schwerpunktpraxis	Peter	Schmidt
Norderstedt	Hämato-onkologisches Zentrum	Suna	Hentschke
		Rüdiger	Hoffmann
Nürnberg	Klinikum Nord	Christiane	Falge
		Hannes	Wandt
		Martin	Wilhelm
Oldenburg	Klinikum	Claus-H.	Köhne
		Cornelia	Schweiger
	Onkologische Praxis	Daniel	Reschke
		Iris	Zirpel
Olpe	Martinus-Hospital	Meinhard	Sauer
		Günter	Lenk
	Onkologische Schwerpunktpraxis	Clemens	Müller-Naendrup
		Hartmut	Eimermacher
Osnabrück	Paracelsus Klinik	Stefan	Frühauf
Penzberg	Städtisches Krankenhaus	Kurt	Ranft
Pforzheim	Klinikum	Lorenz	Theilmann
		Bettina	Sandritter
	MVZ am Siloah St. Trudpert Klinikum	Yves	Dencausse
Pinneberg	Onkologische Praxis	Gerold	Baake

German CML-Study Group and SAKK
(Participants of the CML-Study IV)

Recklinghausen	Elisabeth Krankenhaus	Peter R.	Ritter
		Otto	Kloke
		Dietmar	Wacker
Regensburg	Krankenhaus Barmherzige Brüder	Michael	Schenk
		Ernst-D.	Kreuser
		Anke	Schlenska-Lange
Regensburg	Schwerpunktpraxis für Hämatologie und Onkologie	Robert	Dengler
	Universitätsklinikum	Wolfgang	Herr
		Matthias	Edinger
		Reinhard	Andreesen
		Stefan	Krause
Remscheid	Sana Klinikum	Artur	Wehmeier
Reutlingen	Klinikum am Steinenberg	Bernd	Braun
		Eberhard	Günther
		Alexander	Wacker
Rosenheim	Internistische Gemeinschaftspraxis	Rudolf	Pihusch
Rüdersdorf	Immanuel Klinik	Kerstin	Stahlhut
Rüsselsheim	Internistische Schwerpunktpraxis	Michael	Baldus
Saarbrücken	CaritasKlinikum St. Theresia	Axel	Matzdorff
		Gunther	Pollmeier
Schwäbisch Gmünd	Klinikum	Wolfgang	Grimminger
		Holger	Hebart
Schwäbisch Hall	Diakonie-Krankenhaus	Thomas	Geer
Siegen	Kreisklinikum	Stefan	Schanz
		Christa	Jürß
	St. Marien-Krankenhaus	Winfried	Gassmann
Sigmaringen	Kreiskrankenhaus	Karlheinz	Seitz
		Gabriele	Käfer
St. Gallen (Switzerland)	Kantonsspital	Thomas	Cerny
		Urs	Hess
Stadthagen	Hämatologische & Onkologische Praxis	Constanze	Priebe-Richter
		Özlem	Stange-Budumlu

German CML-Study Group and SAKK
(Participants of the CML-Study IV)

Straubing	MVZ Onkologie am Klinikum Straubing	Matthias	Demandt
		Gernot	Freunek
Stuttgart	Diakonie-Klinikum Stuttgart	Else	Heidemann
		Joachim	Kaesberger
		Rudolf	Mück
	Katharinenhospital	Gerald	Illerhaus
		Jan	Schleicher
	Marienhospital	Claudio	Denzlinger
	Praxis für Onkologie und Hämatologie	Heinrich	Fiechtner
		Gregor	Springer
	Bürgerhospital	Hans-G.	Mergenthaler
		Dietmar	Hoffmann
Teupitz	St. Hedwig-Krankenhaus	Christian	Boewer
Triberg	Asklepios Klinik	Gerhard	Adam
		Christoph	Zeller
Trier	Internistische Gemeinschaftspraxis	Hans-P.	Laubenstein
		Bernhard	Rendenbach
	Klinikum Mutterhaus der Borromäerinnen	Michael	Clemens
		Ali-R.	Waladkhani
Troisdorf	Hämatologisch-Onkologische Schwerpunktpraxis	Helmut	Forstbauer
Tübingen	Internistische Praxis	Swen H.	Jacki
	Universitätsklinikum	Lothar	Kanz
		Martin	Sökler
		Christiane	Dorn
		Ulrike	Bross-Bach
Ulm	Universitätsklinikum	Hartmut	Döhner
		Frank	Stegelmann
Velbert	Gemeinschaftspraxis für Hämatologie und Onkologie	Naser	Kalhori
		Werner	Langer
		Arnd	Nusch
Verden	Praxis für Innere Medizin	Freerk	Müller
Waldbröl	Kreiskrankenhaus	Stefan	Brettner

German CML-Study Group and SAKK
(Participants of the CML-Study IV)

Weiden	Hämatologische Praxis	Johann	Weiß
Wendlingen	Praxis für Innere Medizin	Torsten	Kamp
Wesel	Schwerpunktpraxis für Hämatologie / Onkologie	Claire	Schadeck-Gressel
Wiesbaden	Deutsche Klinik für Diagnostik	Rainer	Schwerdtfeger
	Praxis für Hämatologie und Onkologie	Klaus M.	Josten
		Ortwin	Klein
Wuppertal	Klinikum	Aruna	Raghavachar
	Praxis für Hämatologie und internistische Onkologie	Werner	Fett
	Praxis für Hämatologie und internistische Onkologie	Heribert	Strotkötter
Würselen	Hämatologisch-Onkologische Praxis	Christoph	Maintz
		Matthias	Groschek
Würzburg	Hämatologisch-Onkologische Praxis	Rudolf	Schlag
Würzburg		Björn	Schöttker
	Universitätsklinikum	Hermann	Einsele
		Volker	Kunzmann
		Marie-E.	Goebeler
Zürich (Switzerland)	Onkozentrum Klinik im Park	Jürg	Gmür
Zwickau	Praxis für Innere Medizin	Wolfgang	Elsel
